# Implementation of an Enzyme Membrane Reactor to Intensify the α-*O*-Glycosylation of Resveratrol Using Cyclodextrins

**DOI:** 10.3390/ph14040319

**Published:** 2021-04-01

**Authors:** Irina Ioannou, Eduardo Barboza, Gaëlle Willig, Thomas Marié, Andreïa Texeira, Pierre Darme, Jean-Hugues Renault, Florent Allais

**Affiliations:** 1 URD ABI (Industrial Agro-Biotechnologies), CEBB, AgroParisTech, 51110 Pomacle, France; Eduardo.barboza@agroparistech.fr (E.B.); gaelle.willig@agroparistech.fr (G.W.); thomas.marie90@gmail.com (T.M.); teixeirandreia@gmail.com (A.T.); 2 Université de Reims Champagne Ardenne, ESCAPE EA 7510, 51097 Reims, France; pierre.darme@univ-reims.fr; 3 Université de Reims Champagne Ardenne, CNRS, ICMR UMR 7312, 51097 Reims, France

**Keywords:** process intensification, enzyme membrane reactor, enzymatic *O*-glycosylation, cyclodextrins, resveratrol

## Abstract

The *O*-glycosylation of resveratrol increases both its solubility in water and its bioavailability while preventing its oxidation, allowing a more efficient use of this molecule as a bioactive ingredient in pharmaceutical and cosmetic applications. Resveratrol *O*-glycosides can be obtained by enzymatic reactions. Recent developments have made it possible to selectively obtain resveratrol α-glycosides from the β-cyclodextrin–resveratrol complex in water with a yield of 35%. However, this yield is limited by the partial hydrolysis of the resveratrol glycosides produced during the reaction. In this study, we propose to intensify this enzymatic reaction by coupling the enzymatic reactor to a membrane process. Firstly, membrane screening was carried out at the laboratory scale and led to the choice of a GE polymeric membrane with a cut-off of 1 kDa. This membrane allowed the retention of 65% of the β-cyclodextrin–resveratrol complex in the reaction medium and the transfer of 70% of the resveratrol α-*O*-glycosides in the permeate. In a second step, this membrane was used in an enzymatic membrane reactor and improved the yield of the enzymatic glycosylation up to 50%.

## 1. Introduction

Resveratrol is a polyphenol belonging to the stilbene family and is found in some fruits such as grapes, blackberries or peanuts. It has been shown that resveratrol consumption has beneficial effects on human health. Resveratrol is extensively used in cosmetic formulations as an antioxidant and anti-aging compound thanks to its capacity to (i) scavenge UV-induced free radicals, (ii) stimulate the proliferation of fibroblasts and (iii) increase the concentration of collagen III [1,2]. In medicine, anticancer effects on pancreatic cells have been demonstrated [3]. Moreover, resveratrol has the ability to quench the free radicals believed to be the initiators of neuronal degeneration related to Alzheimer disease [4]. The prevention of diabetes, kidney failure or cardiovascular disease has also been demonstrated [5]. However, some limitations in the use of resveratrol in the pharmaceutical, food and cosmetic fields have been shown [6]. Indeed, the results of in vitro studies have not been confirmed by those obtained in vivo. This can be explained by a very low bioavailability of resveratrol, which leads to a rapid elimination of the molecule in the body, as well as its low solubility in water [7]. The concentrations measured in the blood plasma are thus lower than those required to have a significant effect [8]. Nature has already solved this issue, as resveratrol is mainly found in vegetal matrix in its β-*O*-glycoside form, also known as piceid. *O*-Glycosylation not only allows an increase in resveratrol water solubility and bioavailability, but it also prevents its oxidation and oligomerization [9,10]. Resveratrol *O*-glycoside derivatives can be obtained through chemical synthesis or enzymatic reaction. While chemical synthesis leads to a large amount of chemical waste due to the need of an activating group at the anomeric position as well as protection/deprotection steps, most of the enzymatic reactions previously developed-involving phosphorylase, cyclodextringlucanotransférase (CGTase) or glycosidase use hazardous organic solvents such as DMSO or acetone [11]. New developments in biocatalytic glycosylation have thus been attempted to overcome these drawbacks. Recently, resveratrol α-*O*-glycosides were obtained selectively from the β-cyclodextrin–resveratrol complex in water with a yield of 35% [12]. This optimal yield was achieved by studying, through a Design of Experiments (DoE), the effect of five parameters (CGTase and cyclodextrin concentrations, cyclodextrin/resveratrol ratio, pH and temperature). However, enzymatic processes can suffer from substrate and/or product inhibition or adverse equilibria. For CGTase, these disadvantages are due to its capacity to catalyze four types of reaction: cyclization, coupling, disproportionation and hydrolysis [13,14]. Moreover, the transglycosylation can release some types of sugars, which can be used by the enzyme and inhibit glycoside production [14]. Research efforts have been directed to these disadvantages to some extent. Different strategies from the literature for enzymatic process intensification have been reviewed [15]. Different techniques were listed: enzyme membrane reactors (EMR), membrane contactors, cascade reactions and sorption. These technologies can be used to intensify enzymatic processes, resulting in an increase in the product yield, process productivity, enzyme stability and/or process sustainability [16]. The originality of this paper is to implement and optimize a membrane process making it possible to increase the productivity of an enzymatic reaction, for the glycosylation of resveratrol using cyclodextrins, already published. Indeed, the main limitation of the CGTase-catalyzed α-*O*-glycosylation reaction is the hydrolysis of the resveratrol α-*O*-glycosides during the reaction. This hydrolysis is favored over time and decreases glycosylation yield [12].

Herein, to avoid this side-reaction, in-stream resveratrol α-*O*-glycoside removal by using an enzyme membrane reactor has been investigated. This strategy of intensification would enable an increase in the reaction yield and improve the sustainability of α-*O*-glycosylation processes. A membrane screening, according to their cut-off point, was first performed on a model solution. Then, the selected membranes were implemented with the aforementioned CGTase-catalyzed α-*O*-glycosylation reaction as an enzyme membrane reactor.

## 2. Results

The intensification of the previously optimized enzymatic glycosylation [12] consists of three steps: (i) the determination of the feasibility of the selective filtration, (ii) the choice of the membrane allowing the best separation between the reaction medium and the α-*O*-glycosides, and (iii) the implementation of the enzyme membrane reactor. The first step was conducted on a model solution containing commercially available natural 3-*O*-β-D glycoside of resveratrol (i.e., piceid) whereas the second one used a real medium. 

### 2.1. Proof-of-Concept of the Selective Filtration

**Figure 1:**
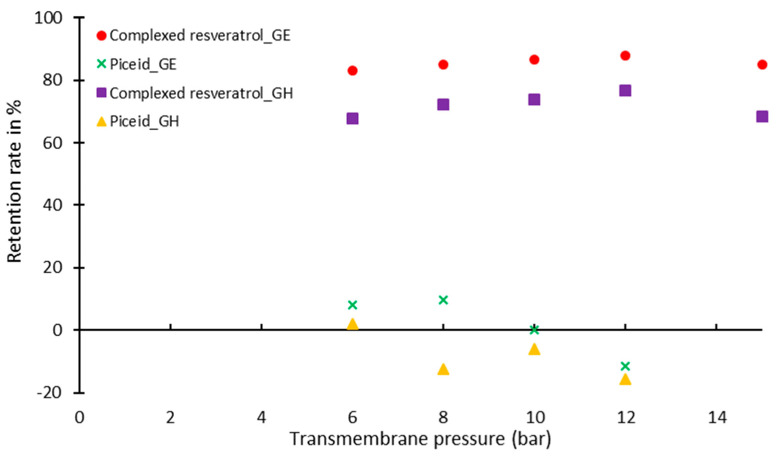
Retention rates according to the transmembrane pressure for GE and GH membranes.

The proof-of-concept of the selective filtration of a β-cyclodextrin–resveratrol inclusive complex (named complexed resveratrol) and piceid solutions was demonstrated by using two membranes, GE and GH, whose cut-offs were 0.9 and 1.4 kDa, respectively. The retention rates as a function of the transmembrane pressure are shown in Figure 1.

Low retention rates (8.6% for GE and 2.2% for GH, at 6 bar transmembrane pressure) were obtained for the piceid solution while high retention levels (83.2% for GE and 67.6% for GH, at 6 bar transmembrane pressure) were obtained for the complexed resveratrol. The negative values obtained can be explained by the rapid elimination of piceid in the permeate. Negative values have already been observed in the literature [17,18].

### 2.2. Choice of the Membranes According to the Screening on Model Solution

#### 2.2.1. Results of the Membrane Screening

Membrane screening was then performed on a model solution composed of β-cyclodextrin (20 g, 17.6 mmol), resveratrol (1 g, 4.3 mmol) and piceid (0.6 g, 1.5 mmol) dissolved in 400 mL of phosphate buffer. A 4/1 β-cyclodextrin/resveratrol ratio was chosen as it was the ratio used for the reaction in the batch mode in Marié et al.’s procedure [12]. Cut-off thresholds ranging from 0.4 to 5 kDa were tested. Table 1 indicates the retention rates for the piceid for the different tested membranes. 

**Figure 2:**
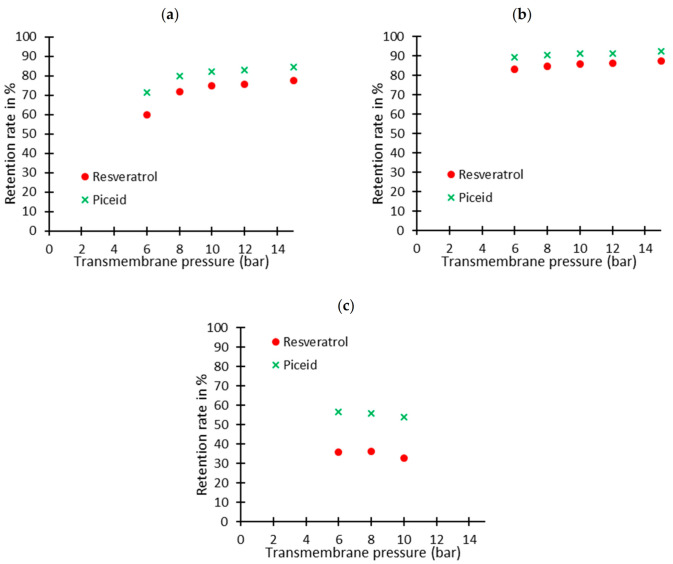
Retention rates for complexed resveratrol and piceid with regards to the transmembrane pressure for (**a**) GE, (**b**) GH and (**c**) PT membranes.

In these conditions—β-cyclodextrin in excess, 6 bar transmembrane pressure—the retention rates obtained for the piceid were high whatever the membrane cut-off threshold. With a GE membrane, a retention rate of the piceid with no free cyclodextrin in the solution (vide supra) was equal to 8.6%, whereas a value of 71.4% was obtained with this model solution when β-cyclodextrin was available for piceid complexation. At this stage, one can conclude that two different inclusion complexes can be formed: cyclodextrin–resveratrol and cyclodextrin–piceid. For the next steps of our study, the membranes with the lowest piceid retention rates (i.e., the highest cut-off threshold) were chosen (GE, GH and PT) as they could provide the highest selectivity between the β-cyclodextrin–resveratrol inclusive complex and the free piceid. Figure 2 shows the evolution of the retention rates of resveratrol and piceid as a function of the transmembrane pressure.

#### 2.2.2. Determination of the Membrane Permeabilities

The effects of the filtration of the model solution on membrane water permeabilities were then studied for the GE and GH membranes. Table 2 shows the values of $L_{p}$ calculated.

The water permeability of the GE membrane obtained before filtration was higher than the manufacturer’s specification (1.21 L × h^−1^ × m^−2^ × bar^−1^) and the data in the literature (at 25 °C, 1.88 ± 0.088 L × h^−1^ × m^−2^ × bar^−1^) [17], especially because of the temperature used in this study. An increase in temperature leads to a decrease in the dynamic viscosity of water and therefore an increase in permeability, according to Darcy’s law. The temperature can also cause the pores to deform.

The same considerations can be applied to the GH membrane. The manufacturer’s specification for the water permeability of the GH membrane was 3.30 L × h^−1^ × m^−2^ × bar^−1^ and the data developed in the scientific literature were at 25 °C, 3.22 ± 0.17 L × h^−1^ × m^−2^ × bar^−1^; at 23 °C, 2.7 ± 0.5 L × h^−1^ × m^−2^ × bar^−1^; and at 30 °C, 3.8 L × h^−1^ × m^−2^ × bar^−1^ [19,20,21]. According to the manufacturer’s specifications, the permeability of the GE membrane should be lower than that of GH, which is contrary to the data obtained. The composition of each of these membranes is not specified by the manufacturer, so temperature can affect these two membranes differently.

A significant decrease in permeability was noticed for the two membranes (30 and 23% for the GH and GE membranes, respectively), suggesting a phenomenon of membrane fouling not investigated in the framework of this study. Nevertheless, the permeate flow remained above 70% of its initial value, which was acceptable for continuing the study.

### 2.3. Implementation of the Enzyme Membrane Reactor for the Resveratrol Glycosylation 

The dead-end and cross-flow configurations were both tested.

#### 2.3.1. Membrane Separation in Dead-End Configuration 

**Figure 3:**
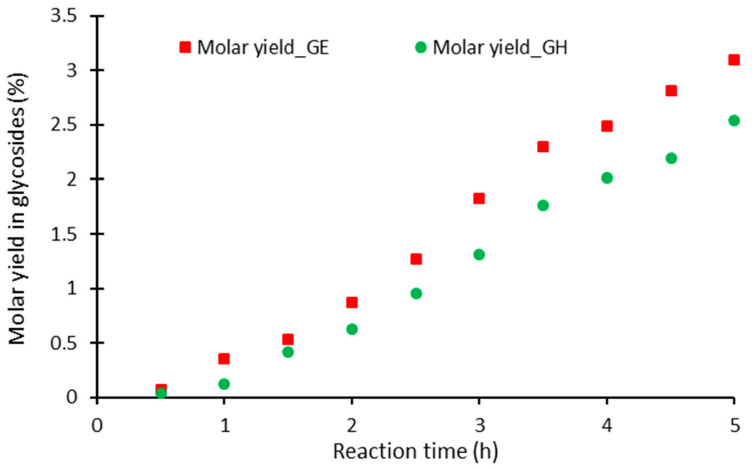
Molar yield of the α-*O*-glycoside of resveratrol in the permeate during the α-*O*-glycosylation reaction with the GE and GH membranes.

The molar yield of the total glycoside forms in the permeate was monitored by quantitative HPLC during the course of the α-*O*-glycosylation reaction (Figure 3).

Low glycoside yields were obtained in the permeate; 3.1% for the GE membrane and 2.5% for the GH, respectively, using the dead-end configuration for the membrane filtration system. In addition, by measuring the α-*O*-glycosides of resveratrol produced in the retentate, the overall yield of the α-*O*-glycosylation reaction was also measured. After a reaction time of 5 h, the overall α-*O*-glycoside yields were 19.4% and 16.9% for the GE and GH membranes, respectively, whereas a global yield of 35% was previously reached in batch operation [12].

#### 2.3.2. Membrane Separation in a Cross-Flow Configuration

**Figure 4:**
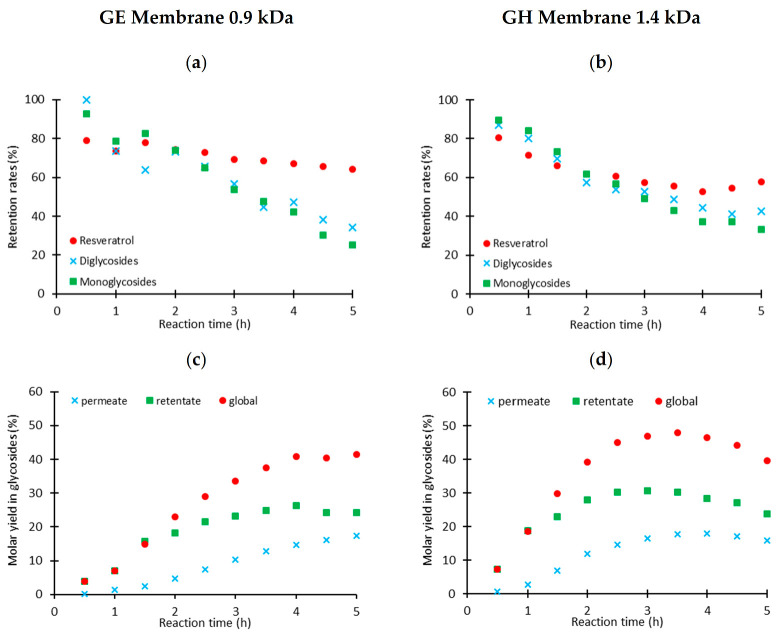
Retention rates (**a**,**b**) and molar yields of glycosides (**c**,**d**) obtained in the coupling of the extractive reaction with GE and GH membranes.

The cross-flow configuration was then evaluated. Figure 4 presents both the retention rates of the resveratrol and its different α-*O*-glycosides and the molar yield of the total α-*O*-glycoside forms in the permeate for the GE and GH membranes (0.9 and 1.4 kDa).

After approximately 2.5 h, the resveratrol retention rate stabilized at 65% with the GE and at 55% with the GH membrane, while the α-*O*-glycoside retention rates continued to decrease, reaching 30 and 40% for the GE and the GH membrane, respectively, after 5 h.

## 3. Discussion

With the yield of the glycosylation reaction being limited by the hydrolysis of the reaction products (resveratrol glycosides), it seems relevant to combine a process for removing the glycosides as soon as they are formed to increase their amounts. 

First, the feasibility of the application of a membrane process to separate piceid from complexed resveratrol was tested. Thus, the retention rates were determined, respectively, from model solutions of the piceid and of the complex. As expected, due to the difference in molar mass (390 g/mol for piceid and 1328 g/mol for complexed resveratrol), low retention rates were obtained for the piceid solution while high retention rates were obtained for the complexed resveratrol. Selective filtration of the reaction medium is therefore possible. 

**Figure 5:**
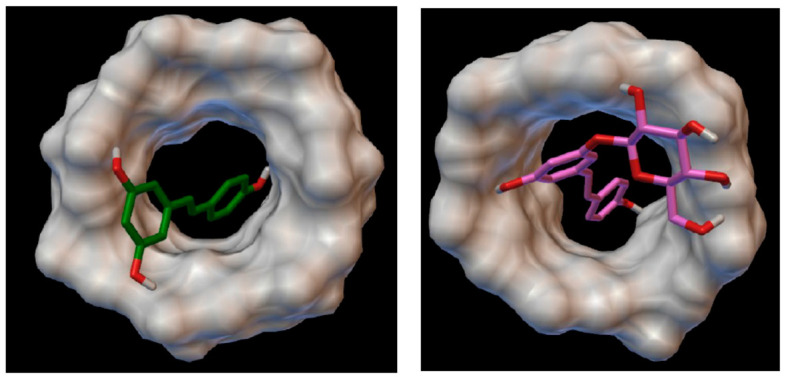
Optimal complex geometries in the case of resveratrol **(left**, binding energy = −5.32 kcal/mol) and piceid (**right**, binding energy = −5.24 kcal/mol).

Secondly, setting up the EMR required determining which membrane was the most suitable for separating the reactants from the reaction products. The experiments were carried out on a model solution reproducing the reaction medium. However, piceid retention rates were higher than those of resveratrol regardless of the membrane and the cut-off threshold. Thus, the resveratrol being in its inclusion complex form in these conditions meant that piceid was also in an inclusion complex form. Modeling experiments using the Autodock4 docking program confirmed that both resveratrol and piceid can be complexed into the β-cyclodextrin cavity through their monophenolic aromatic cycle. Figure 5 shows that the glucose moiety of piceid remained outside the β-cyclodextrin cavity, thus increasing the size of the inclusion complex. 

Two pieces of information can be deduced from these preliminary experiments. Firstly, the model solution led to the formation of an inclusion complex between piceid and β-cyclodextrin, thus preventing the selective retention of resveratrol and the transfer of its *O*-glycosylated form (i.e., piceid) in the permeate. Thus, to eliminate this problem in the case of an enzymatic α-*O*-glycosylation carried out under actual operating conditions, the α-*O*-glycosides of resveratrol synthesized must be continuously removed from the permeate during the reaction. Secondly, the PT membrane has a too-high cut-off threshold and did not provide enough retention of the β-cyclodextrin–resveratrol inclusion complex. This membrane will therefore not be implemented in the enzyme membrane reactor. 

The problem of selectivity between resveratrol and its α-*O*-glycosylated forms could be avoided if the complexation kinetics are sufficiently slow to promote the transfer of free α-*O*-glycosylated forms across the membrane. The hyphenation between the unit operations of enzymatic *O*-glycosylation and membrane filtration has therefore been implemented in dead-end and cross-flow configurations. It is well known that resveratrol can readily oligomerize under certain conditions, and that the resulting oligomers can also be glycosylated. Nevertheless, the findings of Zupancic et al. (2015), who conducted an in-depth study of the stability of resveratrol with regards to pH and temperature, demonstrate that the extent of resveratrol oligomerization in our reaction conditions (pH = 6.2 and T = 60 °C, 2–5 h) is expected to be very limited [22]. It is also noteworthy to mention that the complexation of resveratrol by cyclodextrin stabilizes it, and thus significantly slows down its oligomerization [23]. Even though the presence of glycosides of resveratrol oligomers cannot be ruled out, the HPLC method used herein to calculate the reaction yield takes into account the mono- and di-glycosides of resveratrol only. 

The dead-end configuration allowed the coupling between the two techniques but did not make it possible to optimize the α-*O*-glycosylation yield within a reasonable reaction time. Concerning the cross-flow configuration, as highlighted in Figure 4, no selectivity between resveratrol and its α-*O*-glycosides was observed after a reaction time of two hours, this being due to the formation of inclusion complexes (vide supra) when the cyclodextrin was in a large excess compared to the α-*O*-glycosides. Moreover, as expected, resveratrol glycoside removal as soon as they are produced by using membrane filtration led to an increase in the reaction yield by limiting the hydrolysis phenomenon after 4 h of reaction time [10]. Compared to the 35% yield obtained for the α-*O*-glycosylation reaction without hyphenation with the membrane filtration unit operation, the cross-flow configuration with the GE membrane resulted in a 40% yield after 4 h while a 50% yield was achieved after 3.5 h with the GH membrane. To summarize, intensification of the CGTase-mediated enzymatic α-*O*-glycosylation of resveratrol through its coupling to the membrane process in cross-flow mode allows an increase of 15 percentage units of the global yield of the reaction (from 35 to 50%).

## 4. Material and Methods

### 4.1. Chemical Products

Resveratrol, piceid and β-cyclodextrin were purchased from Tokyo Chemical Industry Europe (TCI Europe). Salts for buffer solutions were acquired from AcrosOrganics. Methanol and acetonitrile with an analytical grade were purchased from Fischer Chemical and VWR, respectively. Toruzymes 3.0 L was bought from Novozymes.

### 4.2. Obtention of the Resveratrol α-glycosides from β-Cyclodextrin–Resveratrol Complex in Water

**Figure 6:**
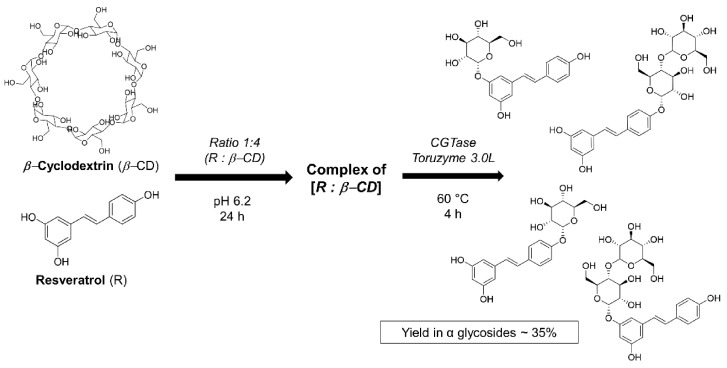
α-*O*-glycosylation reaction of resveratrol with β-cyclodextrin.

The enzymatic α-*O*-glycosylation of resveratrol was carried out in a MES (2-(*N*-morpholino) ethanesulfonic acid) buffer. β-cyclodextrin (70 mg) was mixed with 3.6 mg of resveratrol (4:1 molar ratio) in 4 mL of buffer at pH 6.2 and 80 °C, with the addition of CGTase (Toruzyme 3.0 L) at a concentration of 157 U/g of β-cyclodextrin. The yield of the enzymatic reaction obtained after 2 h was 35% (Figure 6).

### 4.3. Membrane Screening on Model Solution 

#### 4.3.1. Composition of the Model Solution 

The model solution to be implemented for the membrane selection was a mixture of β-cyclodextrin, resveratrol and piceid. β-cyclodextrin (20 g, 17.6 mmol)), resveratrol (1 g, 4.3 mmol) and piceid (600 mg, 1.5 mmol) were dissolved in 400 mL of pH 6.2 phosphate buffer (0.05 mol/L). The solution was then heated for 24 h at 80 °C, in a flask covered with aluminum foil. After 24 h, the solution was cooled to 60 °C before being integrated into the membrane system. A 500 μL aliquot was used for analysis by HPLC. 

#### 4.3.2. Description of the Membrane Process Used to the Screening

**Figure 7:**
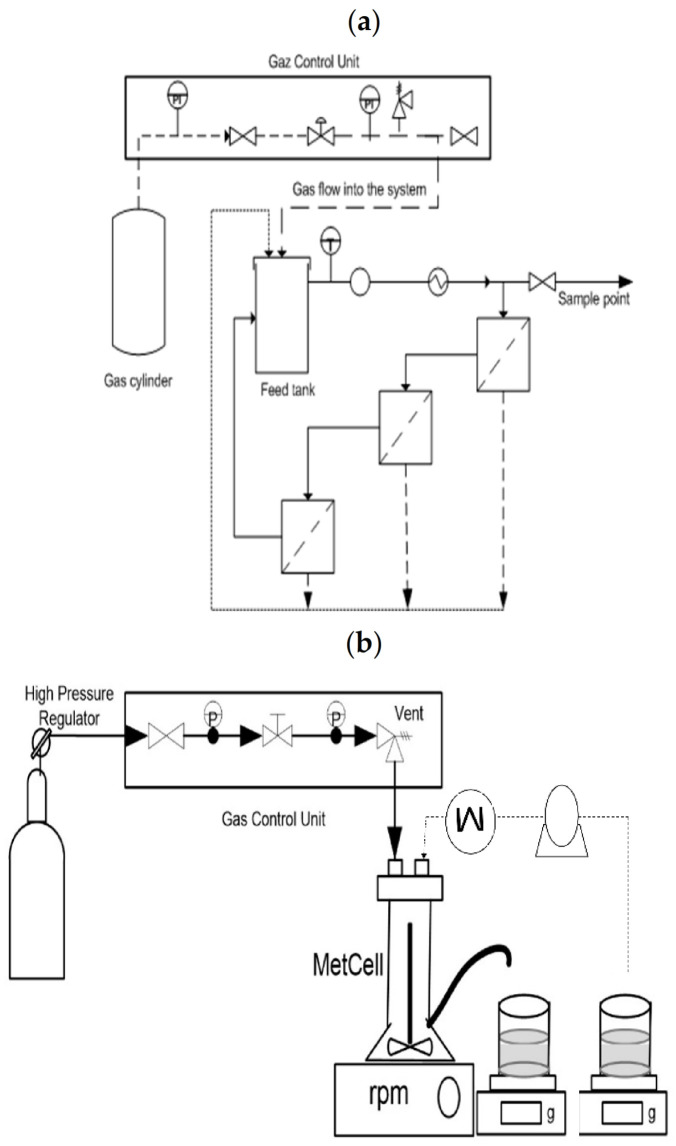
METCell filtration system (**a**) in cross-flow and (**b**) in dead-end configuration.

Laboratory-scale experiments were performed with a METCell filtration system in dead-end and cross-flow configurations with a gas control unit (Evonik Industries, Essen, Germany) (Figure 7). 

Membranes were supported by a porous stainless-steel disc. This system allowed the study of three planar membranes (for an effective area per membrane of 13 cm^2^ and a 600 mL total volume treated) in cross-flow configuration or with the use of a single membrane in dead-end configuration (active surface of 51.4 cm^2^ and a treated volume of 250 mL). The liquid circulated in the system through a recirculation pump, the GC-M23.JF5S.6 (Micropump INC, Vancouver, WA, USA), which promoted the agitation in cross-flow configuration. In dead-end configuration, the agitation was made possible thanks to a cross-head magnetic bar. This system permits implementation from microfiltration to reverse osmosis. The HPLC pump, Model 306 (Gilson, Middleton, WI, USA) allowed working in diafiltration mode and not only in concentration mode or total recirculation. In all cases, the pressure was controlled by a cylinder of inert gas (N_2_) and ranged from 1 to 65 bar. The temperature was controlled by a hot plate, the RCT basic (IKA, Baden-Württemberg, Germany), and a cooler system, the Minichiller300 (Peter Huber Kältemaschinenbau AG, Offenburg, Germany). The amount of permeate was measured gravimetrically, by acquisition (precision 0.1 g) with a balance, the ME4001 (Mettler Toledo, Greifensee, Switzerland).

#### 4.3.3. Protocol for the Membrane Screening 

The membrane choice was guided by both temperature resistance and cut-off thresholds. The membranes had to withstand a temperature of 60 °C, corresponding to the temperature of the glycosylation reaction. Membranes used in the membrane process are listed in Table 3.

The membrane selection was made considering three parameters: the retention rate of resveratrol and glycosides and/or the molar yield in glycosides.

The retention rate assessed the membrane separation efficiency for the target compound. The METcell system was used in total recirculation mode. After compacting the membranes in water at 60 °C, at maximum pressure (15 bar) and until constant permeate flux (usually 30 min), membrane permeability was assessed. Membrane water permeability was determined with ultrapure water at 5 pressures (15, 12, 10, 8 and 6 bar). Tests started with the highest pressure up to the lowest pressure, with a stabilization time of 10 min between each pressure. Membrane water permeability was evaluated before and after the filtration with the solution in order to detect fouling. Each test was realized with a new membrane coupon.

After compacting and evaluating water permeability, the water was removed from the tank and the model solution containing resveratrol, β-cyclodextrin and piceid was placed in the system at 60 °C. The pressure was then adjusted to the maximum pressure used in the membrane test. The system was then tested at 5 pressures; a stabilization of 10 min being performed between each pressure. At each pressure, the permeate flow rate was measured to determine the permeability of the solution. An HPLC sample of 1 mL was taken in the retentate and in the permeate to measure the concentration of resveratrol and piceid, and thus to determine the retention rates as in Equation (1):

(1) $$R=1-\frac{Cp}{Ca}$$

where *R* is the retention rate, *Cp* the concentration of the compound in the permeate and *Ca* the concentration of the compound in the retentate.

The molar yield was calculated when only the permeate could be monitored over time (dead-end configuration). The molar yield of α-*O*-glycosides (*Y*) was given by Equation (2):

(2) $$Y=\frac{N_{\left(glycosides\right)t}}{N_{\left(resveratrol\right)o}}\;\times 100$$

where *N_(glycosides)t_* represents the quantity of α -*O*-glycosides (mol) at a t reaction time and *N_(resveratrol)o_* the initial quantity of resveratrol (mol).

Membrane permeability ($L_{p}$) was defined according to Equation (3):

(3) $$J_{P}=L_{p}\times TMP$$

where $J_{p}$ (L × h^−1^ × m^−2^) and TMP (bar) represent the permeate flux and the transmembrane pressure, respectively. The permeate flux, corresponding to the permeate flow rate per unit area of the membrane, was then determined for 5 pressures. A straight line was obtained by plotting $J_{p}$ according to the transmembrane pressure. $L_{p}$ corresponded to the slope of this straight line.

### 4.4. Description of the Enzyme Membrane Reactor

**Figure 8:**
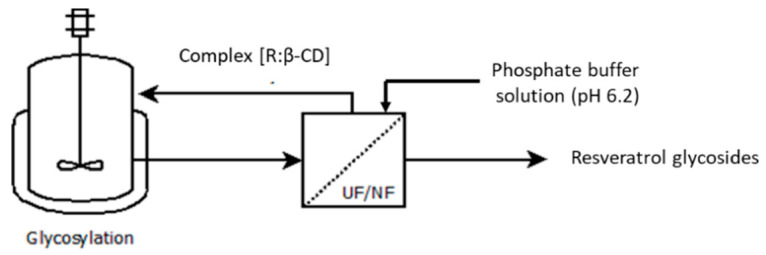
Enzyme membrane reactor used to intensify resveratrol glycosylation.

The enzyme membrane reactor used to intensify the α-*O*-glycosylation of resveratrol consisted of a stirred tank in which enzymatic α-*O*-glycosylation was coupled to a membrane process allowing the passage of small molecules (resveratrol α-*O*-glycosides) (Figure 8).

In this case, the system was configured in a recirculation mode, as the objective was to remove the glycosides from the reaction medium containing the β-cyclodextrin–resveratrol complex in order to intensify the enzymatic α-*O*-glycosylation. The permeate was isolated, and a phosphate buffer solution (pH 6.2) placed in a diafiltration tank was added to the reaction medium. A level probe coupled to a regulation loop made it possible to maintain a constant filtration volume. The membranes tested were those chosen in the screening part. The membrane process was the METCell system used in cross-flow configuration at 6 bar. Regular samples of 1 mL were taken in the permeate stream and in the retentate to determine the retention rates and the process yield.

### 4.5. HPLC Analysis

The contents of resveratrol and α-*O*-glycosides of resveratrol were determined by HPLC on a Dionex Ultimate 3000 system (Dionex Corporation, Sunnyvale, CA, USA). The HPLC system and the method have already been well described [12].

### 4.6. Simulation of the Complexation with β-Cyclodextrin

The coordinates for the β-cyclodextrin were obtained from the X-ray crystal structure of a β-amylase/β-cyclodextrin complex (Protein DataBank code: 1BFN, resolution of 2.07 Å). Optimized β-cyclodextrin, resveratrol and piceid structures were obtained after conformational optimization with the open-source molecular builder and visualization tool Avogadro 1.2.0 (http://avogadro.cc/, 2 June 2020). The MGL Tools 1.5.6 with AutoGrid 4 and AutoDock 4.2 were used to set up and perform docking calculations between resveratrol and piceid as flexible guests, while β-cyclodextrin was used as rigid host. Initially, hydrogen atoms were added into the structure. Then, the partial atomic charges for both resveratrol and piceid as well as for the β-cyclodextrin were calculated using the Gasteiger–Marsili and Kollman methods, respectively [24,25]. A grid box of the size of the β-cyclodextrin was generated using AutoGrid 4 and the ligands were then docked into the β-cyclodextrin cavity by AutoDock 4.2 with the following parameters: run number = 100 and search parameters = lamarkian genetic algorithm. All the other parameters were the default values. The docking analysis was performed using AutoDockTools by combing the docking pose populations and the corresponding binding energies.

## 5. Conclusions

The main objective of the project was to improve the performance of the enzymatic α-*O*-glycosylation of resveratrol by coupling the biocatalytic reaction with a membrane process, to allow the isolation of the α-*O*-glycosides of resveratrol and prevent their hydrolysis. Being more stable and more water-soluble than pristine resveratrol, these glycosides exhibit an improved bioavailability and are thus value-added bioactive ingredients with potential applications in the cosmetic (e.g., antioxidant, anti-aging) and pharmaceutical (e.g., anticancer, diabetes, kidney failure and cardiovascular disease) sectors. After having conducted a membrane screening, two polymeric membranes were chosen (0.9 (GE) and 1.4 kDa (GH)) for testing in both dead-end and cross-flow ultra-filtration configurations. Compared to the classic batch α-*O*-glycosylation of resveratrol in the presence of β-cyclodextrin and CGTase in water (35% yield), the coupling of the reaction with an ultrafiltration process enabled an increase in the reaction yield between 5% and 15%, depending on the membrane used, with a reaction time less than 5 h. The membrane of 0.9 kDa (GE) led to a stable yield of 40% after 4 h of reaction, whereas the membrane of 1.4 kDa (GH) resulted in a maximum yield of 50% at 3.5 h of reaction. The proof-of-concept of selective removal of α-*O*-glycosides of resveratrol to prevent subsequent hydrolysis reported in this study provides interesting improvements of the α-*O*-glycosylation of resveratrol, as yield increases while the reaction time decreases. An optimization step of the operating conditions may further improve this integrated process.

## Figures and Tables

**Table 1:** Piceid retention rates according to the cut-off thresholds of the membranes.

Supplier	Microdyn Nadir	Hydranautics—Nitto	GE	GH	PT
Membrane Cut-Off Threshold (kDa)	0.4	0.72	0.9	1.4	5
Piceid Retention Rates (%)	>90	>90	71.4 (6 bar)	89.1 (6 bar)	56.4 (6 bar)

**Table 2:** Membrane water permeability ($L_{p}$) at 60 °C, before and after filtration.

*L_p_* (L × h^−1^ × m^−2^ × bar^−1^)	Before Filtration	After Filtration	Change
GE	7.34	5.65	−23%
GH	6.73	4.64	−30%

**Table 3:** Characteristics of the used membranes.

Supplier	Type	Membrane	Material	Cut-Off Threshold (Da)	Maximum Temperature and Pressure	pH
General Electrics	Ultrafiltration	GE	Proprietary thin film	900	70 °C/40 b	1–11
		GH		1400	70 °C/27 b	
		PT	Polyethersulfone /polysulfone	5000	70 °C/10 b	
Microdyn Nadir	Nanofiltration	NP030	Polyethersulfone	400	95 °C/40 b	0–14
Hydranautics–Nitto	Nanofiltration	HYDRACoRe 70 pHT	Sulfonated polyethersulfone	720	60 °C/41 b	2–11

## Data Availability

Data are not available.
